# MiRNA profiling of whole trabecular bone: identification of osteoporosis-related changes in MiRNAs in human hip bones

**DOI:** 10.1186/s12920-015-0149-2

**Published:** 2015-11-10

**Authors:** Laura De-Ugarte, Guy Yoskovitz, Susana Balcells, Robert Güerri-Fernández, Santos Martinez-Diaz, Leonardo Mellibovsky, Roser Urreizti, Xavier Nogués, Daniel Grinberg, Natalia García-Giralt, Adolfo Díez-Pérez

**Affiliations:** Musculoskeletal research group, IMIM (Hospital del Mar Medical Research Institute), Red Temática de Investigación Cooperativa en Envejecimiento y Fragilidad (RETICEF), ISCIII, Barcelona, Spain; Departament de Genètica, Centro de Investigación Biomédica en Red de Enfermedades Raras (CIBERER), ISCIII, Universitat de Barcelona, IBUB, Barcelona, Spain; Internal Medicine Department, Hospital del Mar, Universitat Autònoma de Barcelona, Barcelona, Spain; Orthopaedic Surgery and Traumatology Department, Hospital del Mar, Universitat Autònoma de Barcelona, Barcelona, Spain

**Keywords:** Osteoporosis, microRNAs, Osteoblast, Fracture, Epigenetic regulation

## Abstract

**Background:**

MicroRNAs (miRNAs) are important regulators of gene expression, with documented roles in bone metabolism and osteoporosis, suggesting potential therapeutic targets. Our aim was to identify miRNAs differentially expressed in fractured vs nonfractured bones. Additionally, we performed a miRNA profiling of primary osteoblasts to assess the origin of these differentially expressed miRNAs.

**Methods:**

Total RNA was extracted from (a) fresh femoral neck trabecular bone from women undergoing hip replacement due to either osteoporotic fracture (OP group, *n* = 6) or osteoarthritis in the absence of osteoporosis (Control group, *n* = 6), matching the two groups by age and body mass index, and (b) primary osteoblasts obtained from knee replacement due to osteoarthritis (*n* = 4). Samples were hybridized to a microRNA array containing more than 1900 miRNAs. Principal component analysis (PCA) plots and heat map hierarchical clustering were performed. For comparison of expression levels, the threshold was set at log fold change > 1.5 and a p-value < 0.05 (corrected for multiple testing).

**Results:**

Both PCA and heat map analyses showed that the samples clustered according to the presence or absence of fracture. Overall, 790 and 315 different miRNAs were detected in fresh bone samples and in primary osteoblasts, respectively, 293 of which were common to both groups. A subset of 82 miRNAs was differentially expressed (*p* < 0.05) between osteoporotic and control osteoarthritic samples.

The eight miRNAs with the lowest p-values (and for which a validated miRNA qPCR assay was available) were assayed, and two were confirmed: miR-320a and miR-483-5p. Both were over-expressed in the osteoporotic samples and expressed in primary osteoblasts. miR-320a is known to target *CTNNB1* and predicted to regulate *RUNX2 and LEPR,* while miR-483-5p down-regulates IGF2. We observed a reduction trend for this target gene in the osteoporotic bone.

**Conclusions:**

We identified two osteoblast miRNAs over-expressed in osteoporotic fractures, which opens novel prospects for research and therapy.

**Electronic supplementary material:**

The online version of this article (doi:10.1186/s12920-015-0149-2) contains supplementary material, which is available to authorized users.

## Background

Increasing evidence indicates that epigenetic mechanisms are fundamental to the regulation of gene expression. Among them, microRNAs (miRNAs) are small (18–24 nt), non-coding RNAs that negatively regulate gene expression post-transcriptionally by binding to 3’-UTRs of target mRNAs. Deregulation of any of the processes with which miRNAs have been associated –including proliferation, differentiation, and apoptosis-could induce pathophysiological conditions, some of them severe, such as cancer [1].

In bone, miRNAs have been described as key factors regulating bone formation, remodelling, and homeostasis [2, 3]. Several studies have shown that miRNAs are involved in the control of bone cell differentiation and function, as well as lineage commitment and cell progression of mesenchymal stem cells [4]. The understanding of miRNA regulation pathways and the identification of miRNAs involved in skeletal function will be essential to the development of miRNA-based therapeutic strategies for bone diseases. In the field of osteoporosis pathophysiology, very few reports on defects in miRNAs regulation mechanisms are available to date. Li et al. [5] described a mutation in pre-miR-2861 that blocked expression of miR-2861 and caused primary osteoporosis in two related adolescents. In another study, three polymorphisms in the 3’UTR of the *FGF2* gene were genetically associated with bone mineral density (BMD) of the femoral neck [6]. These genetic variants were located in target sequences for miR-146a and miR-146b.

Very recently, Seeliger et al. [7] and Garmilla-Ezquerra et al. [8] performed a microRNA analysis from total bone tissue comparing osteoporotic vs non-osteoporotic bone. Seeliger et al. [7] have identified 6 miRNAs upregulated in osteoporotic fracture patients: miR-21, miR-23a, miR-24, miR-25, miR-100 and miR-125b meanwhile Garmilla-Ezquerra et al. [8] detected miR-187 and miR-518f as differentially expressed between sample groups. Remarkably, both studies detected different miRNAs involved in the osteoporotic physiopathology.

One handicap in the study of miRNAs is their highly variable expression, which depends on cell status (differentiation, proliferation) as well as environment (hormones, cytokines, and other signalling factors). Indeed, profiling miRNA expression in different cell sources of osteoblastic lineage or under different treatments or pathological status results in a variety of bone-regulatory miRNA subsets [9, 10]. Moreover, studies using cells from diverse mammalian species or established cell lines and different culture conditions can identify different miRNAs, making it difficult to compare experiments and results and/or to extrapolate to in vivo physiological conditions.

The aim of this study was to identify miRNAs with altered expression in osteoporotic bone, using an experimental methodology that mimics the physiological conditions. For this purpose, fresh trabecular bone samples from patients with osteoporosis and a recent fracture were compared to those of non-osteoporotic individuals. MicroRNA array analysis was performed in total bone tissue to detect all miRNAs expressed in these samples. Unlike the previous two mentioned reports [7, 8], extremely care was taken in sample selection, resulting in homogenous anthropometric parameters such as age, body mass index (BMI) and gender between groups. Moreover, patients with disorders affecting bone remodelling were excluded from the study.

Furthermore, a complementary array was made from human primary osteoblasts in order to assess which miRNAs from the total bone array were expressed in osteoblastic cells.

## Methods

### Ethics statement

The Clinical Research Ethics Committee of Parc de Salut MAR approved the present research. The approved protocols for obtaining fresh bone (and primary osteoblasts) from hip or knee samples otherwise discarded at the time of orthopaedic surgery were explained to potential study participants, who provided written informed consent before being included in the study.

### Bone samples and RNA extraction

In order to perform the microarray, fresh femoral neck trabecular bone was obtained from 12 postmenopausal women (discovery samples) undergoing hip replacement due to either osteoporotic fracture (*n* = 6) or osteoarthritis in the absence of osteoporosis (*n* = 6) (according to BMD and T-score measurements [mean ± SD]: 0.794 ± 0.074 and −0.25 ± 0.827, respectively). For the replication stage, a new set of samples were collected: 7 postmenopausal women with osteoporotic fracture and 6 with osteoarthritis in the absence of osteoporosis (according to BMD and T-score measurements [mean ± SD]: 0.882 ± 0.158 and - 0.342 ± 1.622, respectively). The osteoarthritic samples were considered the control group because the BMD values classified them as non-osteoporotic. None of the participants had a history of metabolic or endocrine disease, chronic renal failure, chronic liver disease, malignancy, Paget’s disease of bone, malabsorption syndrome, hormone replacement therapy, anti-resorptive or anabolic agents, oral corticosteroids, anti-epileptic drugs, or treatment with lithium, heparin, or warfarin.

Bony fragments were extracted from the transcervical region of the femoral neck for both osteoporotic fracture (OP) and control samples. Total fresh bone samples were cut into small fragments, triple washed in phosphate buffered solution (PBS), and stored at −80 °C until use. RNA extraction was performed at TATAA Biocenter in Gothenburg, Sweden. A piece of tissue was cut out and used in the extraction, following the manufacturer’s instructions for the miRNeasy Mini kit (Qiagen). QIAzol Lysis Reagent was added to the samples and homogenized for 5 min using the TissueLyser system. Chloroform was added to each sample, followed by centrifugation for 15 min at 12000 g. The upper water phase was collected and the extraction continued according to manufacturer’s instructions. The concentration of the purified RNA was analysed on a spectrophotometer (Nanodrop, Thermo Fisher Scientific Inc).

### Human osteoblast culture

Human primary osteoblasts (hOB) were obtained from trabecular bone of postmenopausal women after knee replacement due to osteoarthritis (*n* = 4). Mean age (± SD) was 71.50 ± 9.95 years and BMI was 30.44 ± 2.43. Bony tissue was cut into small pieces, washed in PBS to remove non-adherent cells, and placed on a 140 mm culture plate. Samples were incubated in Dulbecco Modified Eagle Medium (DMEM; Gibco; Invitrogen, Paisley, UK), supplemented with sodium pyruvate (1 mM), L-glutamine (1 mM), 1 % penicillin/streptomycin, 10 % foetal calf serum (FCS), 0.4 % fungizone, and 1 % ascorbic acid. This allowed osteoblastic cells to migrate from the fragments and proliferate. After approximately 3 weeks of culture and before reaching confluence, total RNA was extracted using High Pure RNA Isolation kit (Roche Diagnostics, Indianapolis, USA) according to manufacturer’s instructions. Alkaline Phosphatase activity and osteocalcin gene expression was assessed in order to confirm the osteoblastic phenotype (data not shown).

### microRNA array hybridization and data analysis

Microarray procedure and data analysis were conducted at Exiqon Services, Denmark. The quality of the total RNA was verified by an Agilent 2100 Bioanalyzer profile and 250 ng (total bone) or 750 ng (primary osteoblasts) total RNA from both sample and reference was labelled with Hy3™ and Hy5™ fluorescent dyes, respectively, using the miRCURY LNA™ microRNA Hi-Power Labeling Kit, Hy3™/Hy5™ (Exiqon, Denmark) as indicated by the manufacturer. The Hy3™-labelled samples and a Hy5™-labelled reference RNA sample were mixed pair-wise and hybridized to the miRCURY LNA™ microRNA Array 7th (Exiqon, Denmark), which contains capture probes targeting all human, mouse, or rat microRNAs registered in the miRBASE 18.0. The miRCURY LNA™ microRNA Array Instruction manual and a Tecan HS4800™ hybridization station (Tecan, Austria) were used for this procedure. After hybridization, the microarray slides were scanned and stored in an ozone-free environment (<2.0 ppb ozone) in order to prevent potential bleaching of the fluorescent dyes. The miRCURY LNA™ microRNA Array slides were scanned using the Agilent G2565BA Microarray Scanner System (Agilent Technologies, Inc., USA) and image analysis was carried out with ImaGene® 9 (miRCURY LNA™ microRNA Array Analysis Software, Exiqon, Denmark). The quantified signals were background-corrected (Normexp with offset value 10, see Ritchie et al. 2007 [11]) and normalized using the Lowess (LOcally WEighted Scatterplot Smoothing) global regression algorithm. Following normalization, both unsupervised and supervised data analysis was performed. Principal component analysis (PCA) traditional and matrix plots and heat-map hierarchical clustering were obtained. For PCA plots and the heat map, 50 mircoRNAs were used. They were chosen in an unsupervised manner by taking the normalized and background filtered data and sorting for microRNA expressed above background in all samples and with the largest standard deviation across the entire sample set. For comparison of expression levels, calculated *p-*values were based on moderated t-statistics. The threshold was set at log-fold change (log_2_) > 1.5 and a *p*-value < 0.05 (corrected by Benjamini and Hochberg [BH] multiple testing adjustment [12]).

### Validation and replication of miRNAs by qPCR

MiRNA qPCR and further data analyses were conducted at Exiqon Services, Denmark. Using the miRCURY LNA™ Universal RT microRNA PCR, Polyadenylation and cDNA synthesis kit (Exiqon), 10 ng RNA was reverse-transcribed in 10 μl reactions. cDNA was diluted 100× and assayed in 10 μl PCR reactions according to the protocol for miRCURY LNA™ Universal RT microRNA PCR; each microRNA was assayed once by qPCR on the microRNA Ready-to-Use PCR, custom Pick&Mix panel using ExiLENT SYBR® Green mastermix. Negative controls excluding template from the reverse-transcription reaction were performed and profiled in the same way. Amplification was performed in a LightCycler® 480 Real-Time PCR System (Roche) in 384-well plates, and the amplification curves were analysed using the Roche LC software, both for determination of Cp (by the 2nd derivative method) and for melting curve analysis.

The amplification efficiency was calculated using algorithms similar to the LinReg software. All assays were inspected for distinct melting curves and the Tm was checked to be within known specifications for the assay. Furthermore, assays to be included in the analysis had to be detected with 5 Cps less than the negative control and Cp < 37. Data that did not meet all criteria were omitted from any further analysis.

Normalization was based on the average of the references/normalizer assays detected in all samples. For the present study, the miR-let-7e-5p was used as the normalizer. Hence, the formula to calculate the normalized Cp values is [average Cp(let-7e-5p) – assay Cp (sample)].

Data quality control, unsupervised data analysis, and Student t-test with BH correction were performed (corrected *p*-values < 0.05 were accepted as significant).

### Bioinformatic analyses

Targets for differentially expressed miRNAs were predicted using the following six programs: PicTar (http://pictar.mdc-berlin.de), TargetScan Human (http://www.targetscan.org), miRDB (http://mirdb.org), MiRanda (http://www.microrna.org), DIANA-TarBase (http://diana.imis.athena-innovation.gr), and miRTarBase (http://mirtarbase.mbc.nctu.edu.tw). The DIANA-mirPath web-based computational tool [13] was used to identify molecular pathways potentially altered by the intersection of miRNAs differentially expressed in fractured bone.

### Real-time PCR of osteoblastic markers and CD3

cDNA synthesis was performed using 500 ng of the total extracted RNA from bone samples (OP:*n* = 5; Control:*n* = 6). The product was diluted (1:1) with RNAse-free pure water, and 2 μl was used to determine gene expression of BGLAP (osteocalcin), ALPL (alkaline phosphatase), COL1A1, BMP2, IGF2 and CD3D using qPCR (all reagents were obtained from Applied Biosystems). The results were analysed using ExpressionSuite Software v.1.0.3 (Life Technologies), and the expression levels were calculated against 18S expression and then normalized to an internal sample (relative quantification) using arbitrary units. All qPCR reactions for each sample were performed in triplicate. qPCR for 18S was carried out under the same conditions using an 18S endogenous control (Assay-on-Demand, Applied Biosystems). Gene expression analysis from total RNA was performed twice.

## Results

### Sample description

The anthropometric features of the OP and Control groups were shown in Table 1. Using the Mann–Whitney U test, no statistical differences in these variables were observed between the two groups.

**Table 1:** Patient characteristics

	n	Age	BMI (kg/m^2^)	BMD (g/cm^2^)
		(Mean ± SD)	(Mean ± SD)	(Mean ± SD)
Discovery samples				
Osteoporotic fracture (OP)	6	75.16 ± 3.54	24.38 ± 2.83	Fragility fracture
Control	6	72.5 ± 7.42	26.06 ± 3.25	0.794 ± 0.074
Replication samples				
Osteoporotic fracture (OP)	7	76,29 ± 7,11	27,87 ± 2,57	Fragility fracture
Control	6	73,00 ± 6,63	27,68 ± 2,97	0.882 ± 0.158

*Abbreviations*: *SD* Standard deviation, *BMI* Body mass index, *BMD* Bone mineral density

### Unsupervised analysis of total bone miRNA array

**Fig. 1:**
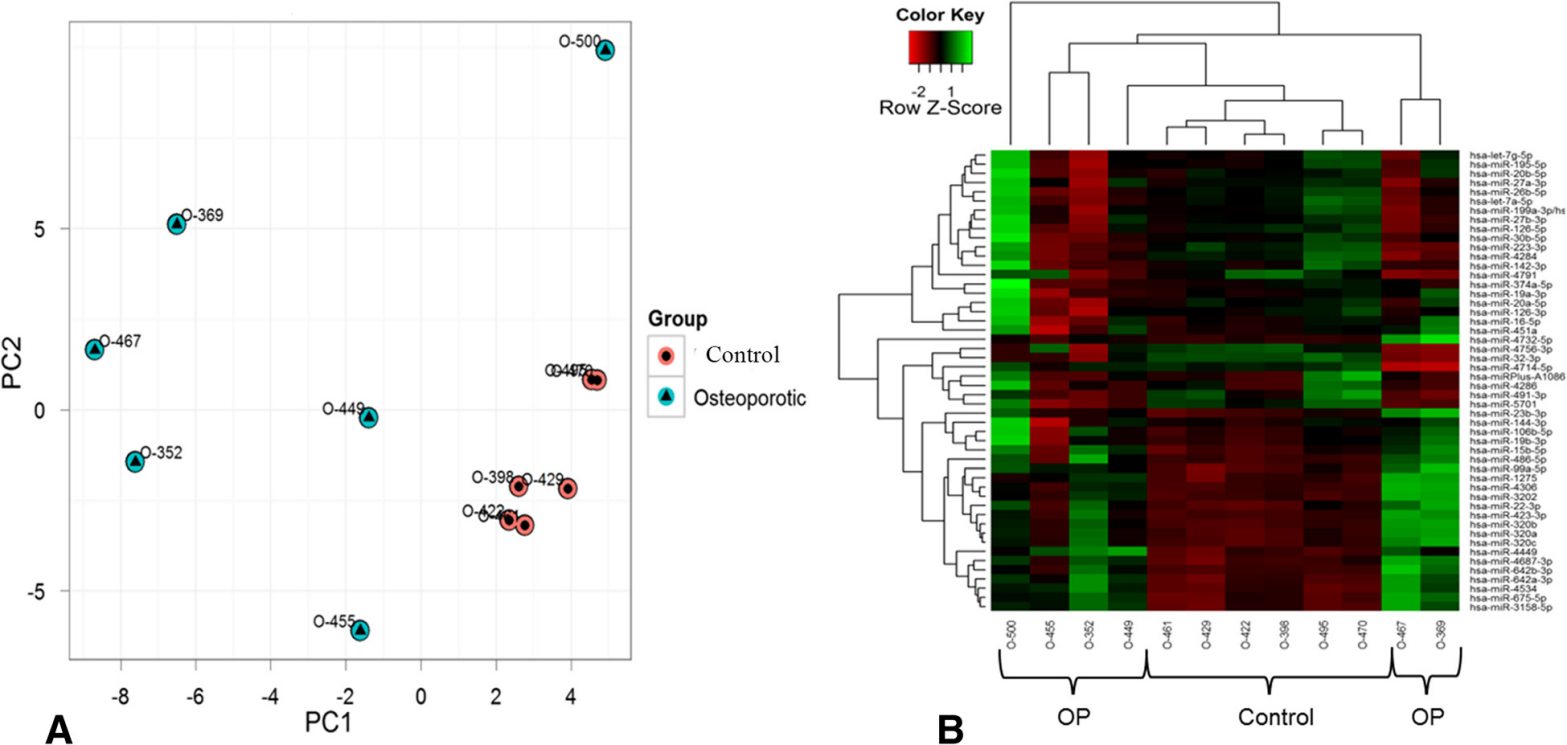
**a** PCA plot and (**b**) heat map diagram. Principal component analysis and clustering was performed on all samples and on the top 50 microRNAs with the highest standard deviation. Normalized log ratio values were used for the analysis. **a** Before the analysis, the features were shifted to be zero centered and scaled to unit variance. **b** Diagram shows the result of a two-way hierarchical clustering of microRNAs and samples. The clustering is done using the complete-linkage method together with the Euclidean distance. Each row represents a microRNA and each column, a sample. The microRNA clustering tree is shown on the left. The colour scale illustrates the relative level of microRNA expression: red, below the reference channel; green, higher than the reference

Trabecular bone samples from patients with osteoporotic fracture were analysed individually in a miRNA array and compared to samples from non-osteoporotic bone. In total, 790 miRNAs were detected when all samples were included. As a first step, an unsupervised analysis of array results based on the expression profile was performed in order to identify variation patterns related to biological or technical factors. A PCA using the 50 miRNAs with the largest variation across all samples was performed to get an overview of how the samples clustered, based on their variance (Fig. 1a). Clustering of non-osteoporotic samples (control group) suggested a homogenous miRNA-expression profile. On the other hand, a visual cluster did not exist in the OP group samples, although they separated from controls according to PC1 (x-axis). Sample O-500, obtained from an OP patient, neither clustered with the other osteoporotic samples nor with the control group, although it behaved as a control according to PC1. This sample was considered an outlier. The heat map diagram, with a clear clustering of control samples and more disperse clustering of osteoporotic samples, corroborates the PCA results (Fig. 1b).

### Comparison of total bone microRNA expression between OP cases and controls

Mean miRNA expression levels were compared between the OP and control groups, excluding sample O-500. This analysis identified a subset of 82 miRNAs (out of the 1932 miRNAs analysed) whose absolute value of log-fold change was larger than 1.5 and adjusted p-value was below 0.05 (Additional file 1: Table S1). Seven of these miRNAs corresponded to SNORDs and three to viruses; all of these were excluded from the validation step.

**Fig. 2:**
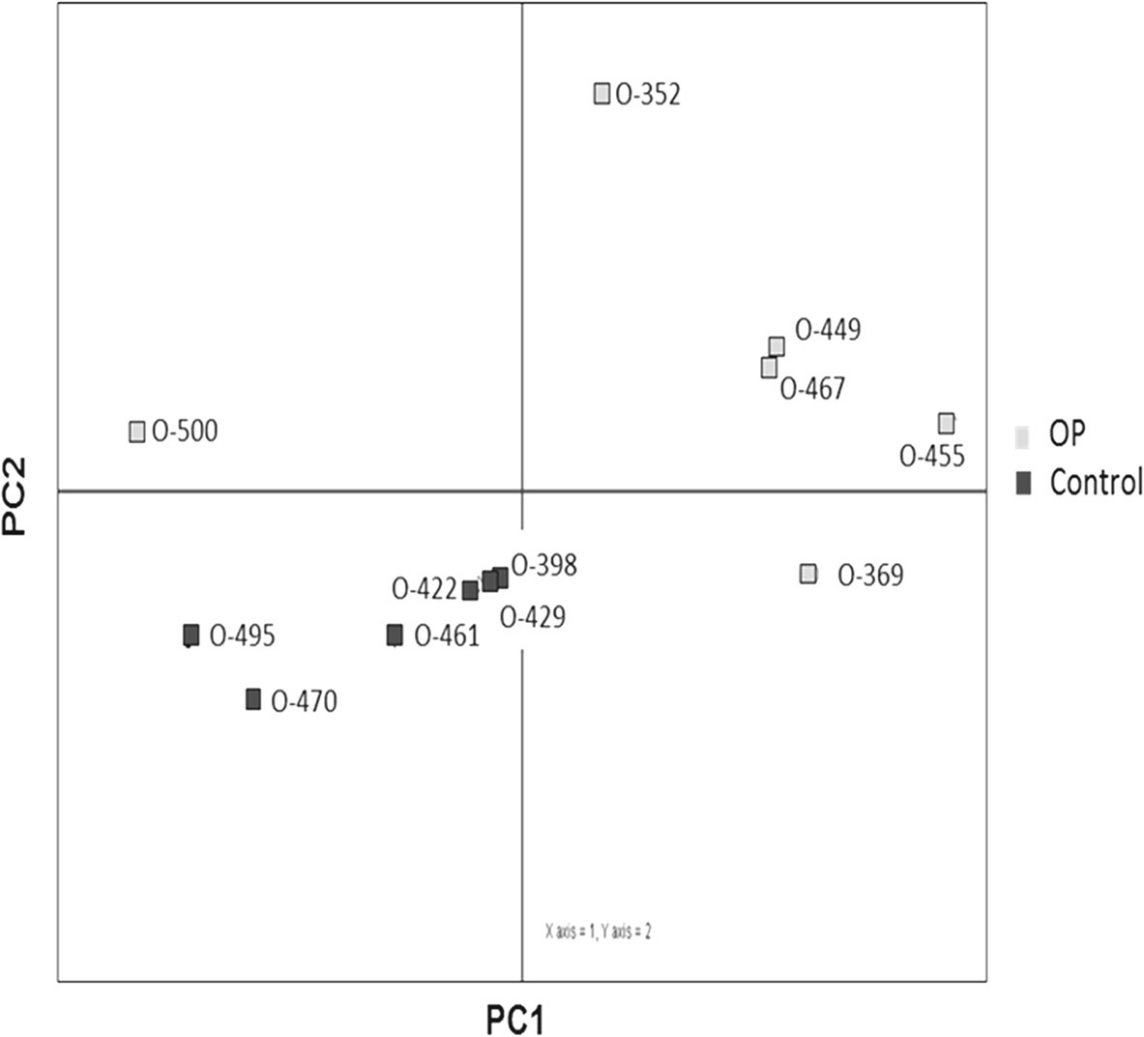
Principal component analysis was performed on all samples. Normalized (dCp) values were used for the analysis. Bone samples from the miRNA array were used for the validation stage (OP:*n* = 6; Control:*n* = 6). Samples are clustered based on their biological group; however, sample O-500 appears to be an outlier

Eight hsa-miRNAs underwent validation in the discovery samples by qPCR (miR-675-5p, miR-30c-1-3p, miR-483-5p, miR-542-5p, miR-142-3p, miR-223-3p, miR-32-3p, and miR-320a) according to the following criteria: available Exiqon probes, the best hits in bone array (signal intensity and significant differences between the groups), and predicted to target genes involved in bone metabolism. The PCA plot of individual qPCR results showed a sample clustering similar to the array results, corroborating the different biological sources of the two sample groups (Fig. 2). Again, sample O-500 could not be clustered with either biological group.

In order to exclude the presence of different amount of hematopoietic cells among bone samples, especially between osteoporotic and control group, the expression of CD3 marker was analysed by qPCR. No significant differences were detected among samples neither between biological groups (Additional file 2: Figure S1). Moreover, this expression was very low compared to CD3 + human T-cells which were FACS sorted from peripheral blood mononuclear cells obtained from buffy coats (data not shown). Hence, differences observed among samples are not due to the blood cells present within bone fragments.

**Fig. 3:**
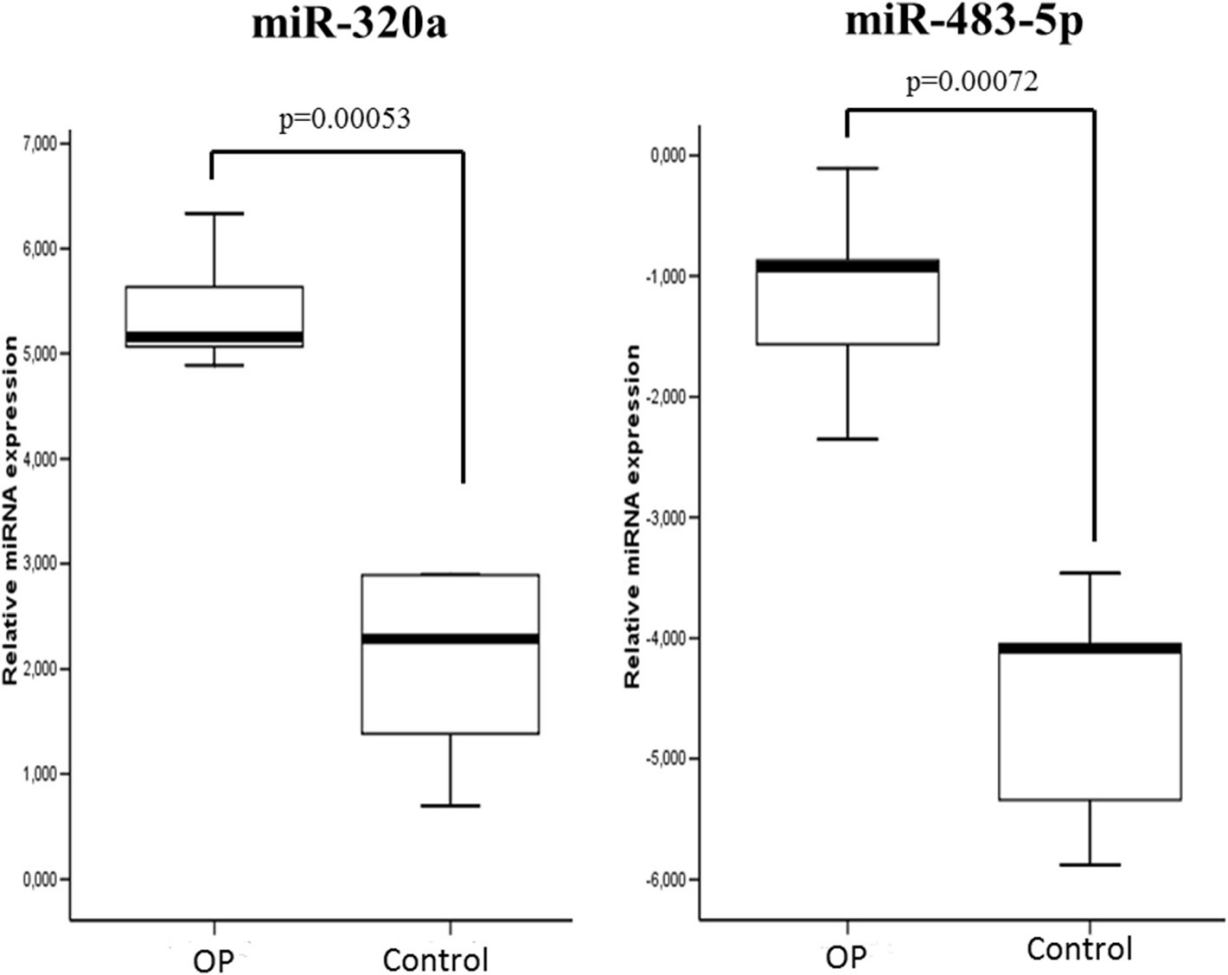
miR-320a and miR-483-5p were validated by qPCR. Relative expression levels of miR-320a and miR-483-5p at the validation stage in patients with osteoporotic fracture (*n* = 5) and controls (*n* = 6). miRNA let-7e-5p was used as a normalizer

After validation, three miRNAs –miR-30c-1-3p, miR-320a, and miR-483-5p– showed significant differences between the OP and control groups (Table 2). However, only miR-320a and miR-483-5p withstood BH multiple-testing correction (Fig. 3).

**Table 2:** qPCR-validated miRNAs that reached significance in the microRNA array

	Average dCp		ddCp			
miR name	Control	OP	LogFC	SD	P	BH adj.
	(*n* = 6)	(*n* = 5)				*p-*value
hsa-miR-320a	2.08	5.42	−3.34	1.88	5.89E-05	5.30E-04
hsa-miR-483-5p	−4.48	−1.16	−3.32	1.98	1.59E-04	7.17E-04
hsa-miR-30c-1-3p	−7.25	−5.50	−1.75	1.28	4.62E-02	6.93E-02
hsa-miR-32-3p	−4.92	−3.76	−1.16	1.36	1.39E-01	6.93E-02
hsa-miR-142-3p	4.39	4.38	0.01	1.07	9.86E-01	6.93E-02
hsa-miR-223-3p	8.05	7.67	0.38	1.57	7.48E-01	8.26E-01
hsa-miR-542-5p	−3.34	−3.22	−0.12	0.73	8.26E-01	8.26E-01
hsa-miR-675-5p	−6.48	−4.87	−1.61	1.16	Not calculated^a^	

^a^miR-675-5p was detected in a low number of samples, which precluded statistical comparison between groups

The replication samples were added in the analysis in order to increase the sample size. The miR-320a (*p* = 0.005) and miR-483-5p (*p* = 0.036) still showed significant differences between biological groups.

### Expression of IGF2 in total bone mRNA from OP cases and controls

**Fig. 4:**
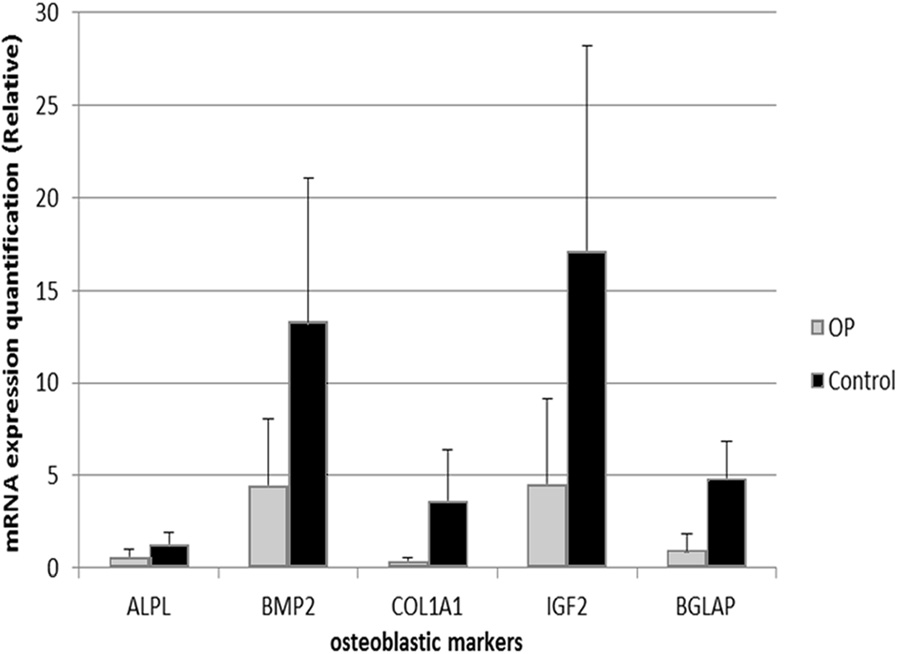
Gene expression quantification of osteoblastic markers by Real-Time PCR in total bone samples. *ALPL, BMP2, COL1A1, BGLAP,* and *IGF2* gene expression was quantified in osteoporotic (OP) samples (*n* = 5) and compared to osteoarthritic (control) samples (*n* = 6). 18S was used as endogenous control. Results are expressed as mean and standard error

Since miRNA miR-483-5p is located within intron 2 of *IGF2,* the expression of this gene was assessed by qPCR to assess its co-expression with miR-483-5p. In the OP samples, *IGF2* was under-expressed compared to control samples (Fig. 4). Thus, *IGF2* did not show the over-expression found for miR-483-5p in the OP group, instead showing a trend in the opposite direction. BMP2, COL1A1, alkaline phosphatase, and osteocalcin gene expression were also evaluated in OP and control samples as disease reference markers. All four showed diminished levels in the osteoporotic bone, as expected (Fig. 4). Although a trend is clearly detected in all genes tested, the limited sample size and experiment replication did not allow sufficient power to reach statistical differences between the biological groups.

### MicroRNA array profiling of cultured primary osteoblasts

**Fig. 5:**
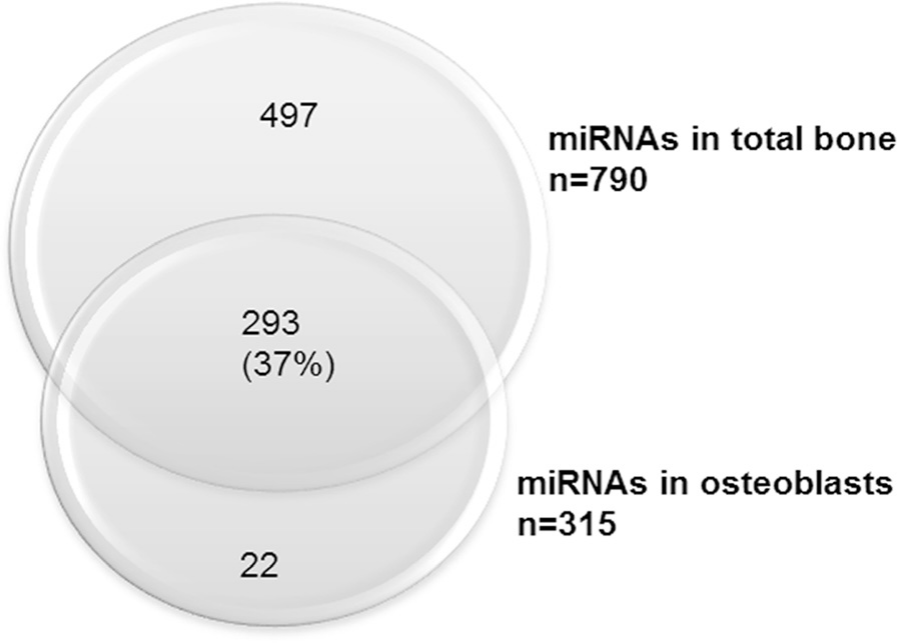
Venn diagram of total bone and osteoblast miRNAs detected in the array

To determine which of the total bone miRNAs were expressed in osteoblasts, miRNA profiling of four hOB samples in passage 0 was performed. A total of 315 miRNAs were detected in osteoblasts, of which 293 (93 %) were also detected in total bone samples (Fig. 5). These 293 osteoblastic miRNAs represent a 37 % of all 790 bone miRNAs. Regarding the 82 miRNAs differentially expressed between control and OP samples, 46 were also detected in the osteoblast array, including miR-320a and miR-483-5p. Interestingly, 22 miRNAs found in the hOB array were not detected in total bone.

### Target prediction and pathway analysis for differentially expressed miRNAs

A comprehensive bioinformatics analysis using six different programs allowed us to predict putative targets for the two differentially expressed miRNAs identified. Targets predicted and validated for miR-320a are involved in a variety of cellular processes, including cell proliferation (SRF, ARPP-19, PDGFD) and apoptosis inhibition (MCL1), signal transduction (MAPK1, SGK, DNER, JAK2), gene expression regulation (PPARGC1A, SP1, CAMTA1, ESRRG), growth factors (IGF1, BMP3 and 6), and hormone, growth factor, and cytokine receptors (LEPR, NR3C1, BMPR1A, RARG, RXRA, IGF1R, IL6R, PTGER3, TFR1). This miRNA is involved in 45 KEGG pathways according to DIANA-mirPath predictions, the most significant being prostate cancer (6.436856e-14), endometrial cancer (4.517902e-10), mTOR signalling pathway (1.336018e-07), and PI3K-Akt signalling pathway (2.347282e-07).

Cell proliferation factors such as SRF and MAPK3 (mirTArBase) are validated targets for the less-studied miR-483-5p. KEGG pathways involving miR-483-5p target genes are mainly ECM-receptor interaction (0.00114789) and N-Glycan biosynthesis (5.118433e-07), as well as PI3K-Akt signalling (0.01153999) and focal adhesion (0.002081914) (DIANA-mirPath).

**Fig. 6:**
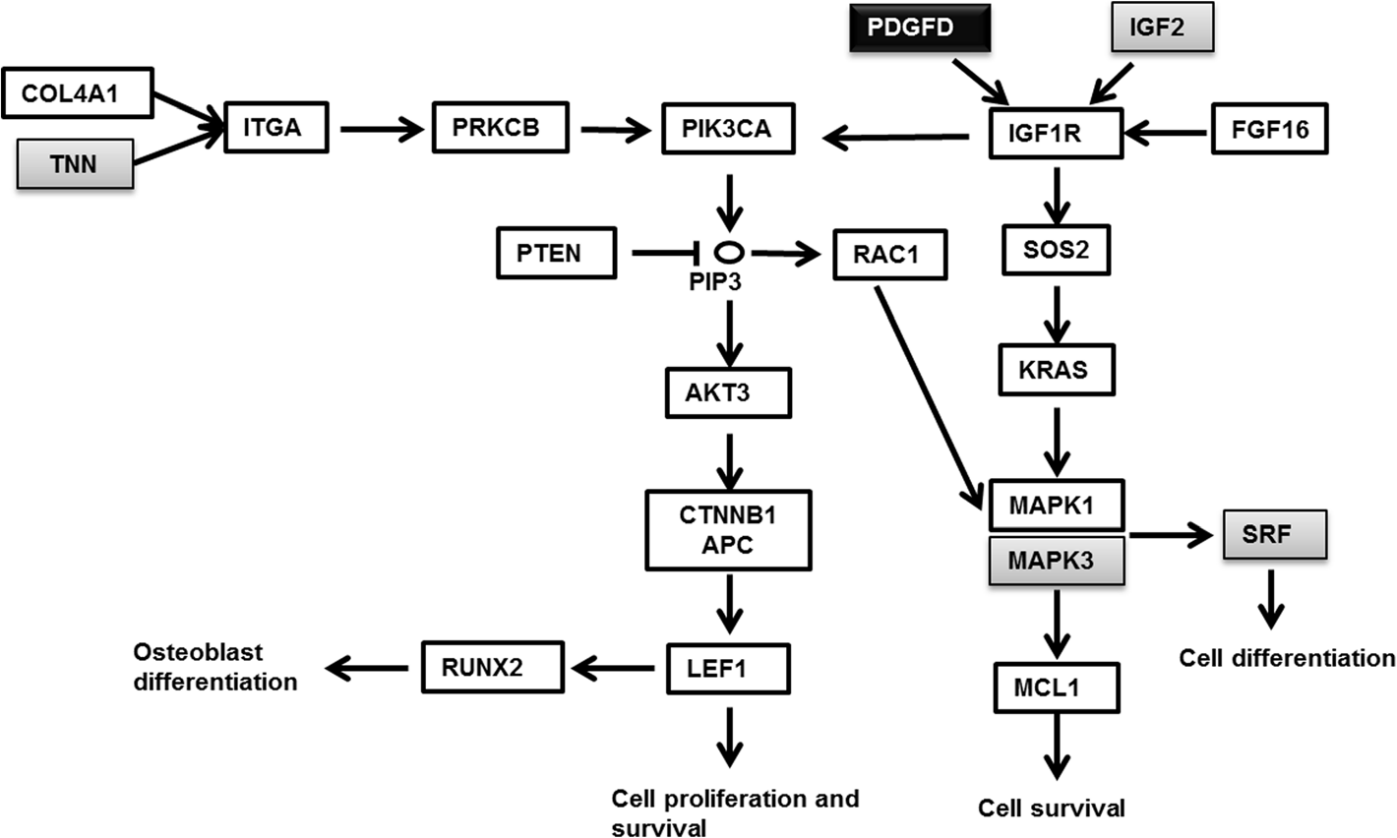
Schematic pathway involving miR320a and miR483-5p target genes. miR-320a target genes are shown in white boxes, miR-483-5p target genes in grey boxes and the black box shows a common target gene for both miRNAs

The intersection pathways involving genes targeted by miR-320a and miR-483-5p are mainly prostate cancer (4.496403e-14), PI3K-Akt signalling (5.614388e-08), and focal adhesion (6.000918e-07). Genes belonging to the mentioned pathways which are targeted by these two miRNAs are shown in Fig. 6.

## Discussion

The present study is focused on the identification of miRNAs with altered expression in osteoporotic bone. Fresh trabecular bone was collected from patients with and without osteoporotic fracture with homogenous anthropometric parameters such as age, BMI, gender or disorders affecting bone remodelling. These accurate selection criteria of samples allowed us to identify, unlike previous studies, miRNAs altered due to osteoporotic fracture specifically. The results obtained can help to identify putative targets for new therapeutic approaches.

The unsupervised analysis of the miRNA profiling from total bone samples showed that osteoporotic bone clearly differed from control samples, which were able to generate a biological cluster. In contrast, osteoporotic samples presented a much more scattered distribution. It is tempting to speculate that the pathophysiology of the osteoporotic disease and ultimately the bone fracture has a heterogeneous aetiology illustrated by different miRNA expression patterns. Working with human samples of fresh bone directly obtained from fractured sites generates higher variability than working with cell lines or isogeneic animal models, but provides a scenario closer to the pathophysiological situation. Hence, our study is based on human bone samples obtained with minimal laboratory manipulation.

In the analysis of differential expression between osteoporotic and control samples, 82 miRNAs reached significance, and after qPCR validation, two miRNAs were significantly over-expressed in osteoporotic samples (miR-320a and miR-483-5p). Both miRNAs were found in the hOB array, suggesting a possible alteration in the osteoblastic cell function. However, since these miRNAs are known to be expressed in a wide variety of cell types, we cannot rule out their expression in other bone cells, and hence a wider spectrum of bone cellular dysfunction. Another miRNA, miR-30c-1-3p, was also over-expressed in osteoporotic samples, although this did not withstand multiple-testing correction. miR-30c-1-3p was not detected in the hOB array. This may suggest a hematopoietic origin for this miRNA since it was reported in macrophages [14] and NK cells [15].

According to our results, 37 % of total bone miRNAs are expressed in bone-forming cells. There are 22 miRNAs detected in the hOB array that were not found in total bone. It may be that hOB cultures, grown in a homogenous and controlled environment, allow for the detection of low-expressed osteoblast-specific miRNAs, which would be diluted in total trabecular bone. Alternatively, *in vitro* conditions of cultured cells may create an artificial environment leading to an altered miRNA expression.

miR-320a, which is conserved in human, mouse, rat, and cow, is encoded within the basal promoter of the cell-cycle gene *POLR3D* in the anti-sense orientation. Interestingly, this miRNA targets the *POLR3D* promoter in cis with perfect complementarity, leading to its direct transcriptional silencing [16]. miR-320a has been extensively studied, both in cancer and in osteoblastic cell function. Some reports indicate that this miRNA may regulate the osteoblast differentiation [17] by targeting key bone-formation genes such as *CTNNB1* (B-catenin) [18] and *RUNX2* [19]. In this context, Hamam et al. [20] observed that miR-320 family (miR-320a, 320b, 320c, 320d and 320e) were upregulated during adipogenesis suggesting the miR-320 family as possible molecular switch promoting adipocytic differentiation of hMSC by targeting *RUNX2, MIB1, PAX6, YWHAH* and *ZWILCH*.

miR-483-5p has been associated with several pathological conditions, including tumours such as adrenocortical and ovarian serous carcinoma [21, 22], cartilage-associated pathologies such as multiple osteochondroma [23] and osteoarthritic chondrocytes [24]. This miRNA has also been detected in human osteosarcoma cells [25]. MiR-483 is encoded within the second intron of the *IGF2* gene. The pre-miRNA generates two mature miRNAs, miR-483-5p and miR-483-3p, both of them present in the bone and hOB profiles of this study, although only miR-483-5p showed significant differences between the control and OP groups. Liu et al. [26] demonstrated that miR-483-5p binds to the 5’-UTR of the major foetal *IGF2* promoter transcript, enhancing the association of the RNA helicase DHX9 to the *IGF2* mRNA and leading to its up-regulation. However, miR-483-5p does not act on promoters that drive adult *IGF2* expression. In mouse Hepa1-6 cells, Ma et al. [27] determined that miR-483-5p is transcriptionally co-expressed with its host gene, *Igf2*, and negatively regulates it. The same pattern of regulation was observed in cartilage samples from old mice and murine osteoarthritic cartilage, in which there were higher levels of miR-483 (orthologous to hsa-miR-483-5p) and lower mRNA levels of *Igf2* [28]. In agreement with these data from the murine system, when we tested *IGF2* mRNA levels in the osteoporotic bone samples, in which miR-483-5p was up-regulated, we observed a trend of reduction relative to controls (see Fig. 4). To our knowledge, this is the first time in which this miRNA that down-regulates *IGF2* is found over-expressed in osteoporotic bone. Osteoblasts synthesize IGF-I and IGF-II, which have mitogenic effects on bone cells and also promote collagen production and matrix apposition [29]. We tested osteoblastic markers in our samples of total fresh bone in order to compare the osteoblastic function between biological groups. Visibly, the gene expression results showed a diminished expression of all tested markers (*BMP2*, *COL1A1*, alkaline phosphatase, and osteocalcin), corroborating the abnormal functionality of osteoporotic bone samples used in this study. However, the limited sample size does not allow sufficient statistical power to determine significant differences between groups in these qPCR results.

When the intersection of the pathways targeted by miR-320a and miR-483-5p were explored, prostate cancer, PI3K-Akt signalling, and focal adhesion emerged as the most significant. These three pathways share many genes: *SOS2, IGF1R, PDK1, PDGFD, PIK3CA, AKT3, PTEN,* and *MAPK1*. Most of them belong to cell proliferation and survival signalling, suggesting a dysfunction in the osteoblastic cell renewal in the osteoporotic bone.

Regarding miR-30c-1-3p (miR-30c-1*), it is an intronic miRNA encoded within the *NFYC* (nuclear transcription factor Y, gamma) gene. Although it did not withstand multiple-testing correction, it is worth noting that the analysis of targeted genes for this miRNA showed that Wnt signalling is the most significantly affected pathway, containing up to eight targeted genes. The key role of this pathway in bone remodelling is well known [30].

Very recently, Seeliger et al. [7] and Garmilla-Ezquerra et al. [8] performed a microRNA analyses from total bone tissue comparing osteoporotic vs non-osteoporotic bone, resulting in different miRNA expression pattern compared to our findings. Remarkably, these two previous studies have several features that can explain these discrepancies. Among them are the diverse experimental approaches, arrays, and sample sizes used. Moreover, these previous studies compared patients with non-homogeneous anthropometric or clinical characteristics, such as age, sex, BMI, and endocrinology disorders (diabetes mellitus), which are essential for the regulation of bone metabolism. Therefore, it might be that their findings had been interfered by these external conditions while we stressed on the absence of concomitant diseases or external factors that potentially could induce noise.

An important strength of our study is the extremely careful control of potentially confounding characteristics between cases and controls in terms of age, sex, BMI and metabolic diseases highly prevalent in this population as diabetes. These strict inclusion criteria explain the relatively small sample size in our study, compared to other similar works where samples are from patients with more heterogeneous characteristics. Therefore, the miRNAs detected in our study can be more reliably considered involved in the osteoporotic phenotype. On the other hand, a possible limitation of our approach is that the non-fractured patients used as controls were osteoarthritic and other bone abnormalities may be occurring in this group. Due to obvious ethical reasons the collection of fresh bone from joint samples of healthy individuals is not allowed. However, in an attempt to minimize this potential caveat, we were careful in obtaining the bone samples from a location distant from the interface between bone and cartilage and, therefore, as far away as possible from the osteoarthritic lesion.

At this stage of miRNA discovery, results from our study and others offer an important breakthrough for the understanding of miRNA biology and pathology in bone. While serum analysis could be interesting to find biomarkers, extensive analyses of osteoporosis-related miRNAs in bone tissue is required to better understand the role of miRNAs in the disease and to explore the potential to design local therapeutic approaches.

## Conclusions

We have identified two miRNAs over-expressed in bone samples from patients with osteoporosis and fracture compared to patients with osteoarthritis in the absence of osteoporosis. The expression of both miRNAs has been detected in osteoblasts. Both are putative regulators of key genes required for bone metabolism. Whether these altered miRNAs are a cause or a consequence of the disease remains to be elucidated. Nonetheless, they might offer promising potential as therapeutic targets in osteoporosis.

### Availability of supporting information

The data sets supporting the results of this article are available in the NCBI GEO repository and are accessible through GEO accession number GSE74209 for whole trabecular bone miRNA array (http://www.ncbi.nlm.nih.gov/geo/query/acc.cgi?acc=GSE74209) and GSE74211 for primary human osteoblasts miRNA array (http://www.ncbi.nlm.nih.gov/geo/query/acc.cgi?acc=GSE74211).

## Additional files

Additional file 1: Table S1. Significant differentially expressed miRNAs between osteoporotic and control bone samples. Logarithmic fold change (logFC); adjusted P. Value (adj.P. Val); standard deviation (SD). (DOCX 38 kb) Additional file 2: Figure S1. Gene expression quantification of CD3 marker by Real-Time PCR in total bone samples. OP samples (*n* = 3); Osteoarthritic (control) samples (*n* = 6). Results are expressed as mean of relative expression and standard deviation. A) Gene expression in each bone sample; B) Gene expression comparison between biological groups. (DOCX 59 kb)
