# Erratum to: MiRNA profiling of whole trabecular bone: identification of osteoporosis-related changes in MiRNAs in human hip bones

**DOI:** 10.1186/s12920-017-0272-3

**Published:** 2017-05-23

**Authors:** Laura De-Ugarte, Guy Yoskovitz, Susana Balcells, Robert Güerri-Fernández, Santos Martinez-Diaz, Leonardo Mellibovsky, Roser Urreizti, Xavier Nogués, Daniel Grinberg, Natalia García-Giralt, Adolfo Díez-Pérez

**Affiliations:** 1 Musculoskeletal research group, IMIM (Hospital del Mar Medical Research Institute), Red Temática de Investigación Cooperativa en Envejecimiento y Fragilidad (RETICEF), ISCIII, Barcelona, Spain; 2 Departament de Genètica, Centro de Investigación Biomédica en Red de Enfermedades Raras (CIBERER), ISCIII, Universitat de Barcelona, IBUB, Barcelona, Spain; 3 Internal Medicine Department, Hospital del Mar, Universitat Autònoma de Barcelona, Barcelona, Spain; 4 Orthopaedic Surgery and Traumatology Department, Hospital del Mar, Universitat Autònoma de Barcelona, Barcelona, Spain

## Erratum

Funding for the work carried out in this article [1] was supported by grant FIS PI10/01537 and the Red Temática de Investigación Cooperativa en Envejecimiento y Fragilidad (RETICEF RD12/0043/0022) (Carlos III Health Institute, Science and Innovation Ministry), and FEDER funds.

## References

[1] De-Ugarte L, Yoskovitz G, Balcells S, Güerri-Fernández R, Martinez-Diaz S, Mellibovsky L, Urreizti R, Nogués X, Grinberg D, García-Giralt N, Díez-Pérez A. MiRNA profiling of whole trabecular bone: identification of osteoporosis-related changes in MiRNAs in human hip bones. BMC Medical Genomics. 2015;8:75. doi:10.1186/s12920-015-0149-2. 10.1186/s12920-015-0149-2 PMC4640351 26555194

