# Supplementary material for: MiRNA profiling of whole trabecular bone: identification of osteoporosis-related changes in MiRNAs in human hip bones
Source: BMC Med Genomics. 2015 Nov 10;8:75. doi: 10.1186/s12920-015-0149-2 (PMC4640351; doi:10.1186/s12920-015-0149-2)
Supplement: Additional file 1: Table S1. — Significant differentially expressed miRNAs between osteoporotic and control bone samples. Logarithmic fold change (logFC); adjusted P. Value (adj.P. Val); standard deviation (SD). (DOCX 38 kb) [file 12920_2015_149_MOESM1_ESM.docx]

Table S1. Significant differentially expressed miRNAs between osteoporotic and control bone samples.

|  |  |  |  |  | **Control** | | **OP** | |  |
| --- | --- | --- | --- | --- | --- | --- | --- | --- | --- |
| **miRNA annotation** | **logFC** | **AvgHy3** | **P.Value** | **adj.P.Val** | **mean** | **SD** | **mean** | **SD** | **logFC(abs)** |
| SNORD118 | -3.238 | 9.093 | 3.09E-05 | 1.00E-03 | 1.19 | 0.14 | -2.05 | 1.35 | 3.24 |
| ebv-miR-BART6-3p | 2.447 | 9.752 | 1.46E-05 | 6.47E-04 | -1.99 | 0.52 | 0.46 | 0.74 | 2.45 |
| hsa-let-7a-5p | -1.819 | 7.097 | 6.13E-04 | 6.99E-03 | 2.94 | 0.78 | 1.12 | 0.64 | 1.82 |
| hsa-miR-1185-2-3p | 1.685 | 7.308 | 3.54E-03 | 2.37E-02 | -1.56 | 0.15 | 0.13 | 1.27 | 1.68 |
| hsa-miR-126-5p | -1.540 | 6.851 | 2.93E-03 | 2.11E-02 | 2.42 | 0.55 | 0.88 | 0.95 | 1.54 |
| hsa-miR-1275 | 2.259 | 12.760 | 3.37E-04 | 4.90E-03 | -1.64 | 0.41 | 0.62 | 1.16 | 2.26 |
| hsa-miR-1307-5p | 1.552 | 6.400 | 9.31E-03 | 4.81E-02 | -1.40 | 0.13 | 0.15 | 1.37 | 1.55 |
| **hsa-miR-142-3p** | **-1.850** | **9.022** | **4.44E-03** | **2.77E-02** | **2.14** | **1.22** | **0.29** | **0.52** | **1.85** |
| hsa-miR-1470 | 1.690 | 6.478 | 1.21E-05 | 5.85E-04 | -0.58 | 0.15 | 1.10 | 0.60 | 1.69 |
| hsa-miR-1915-3p | 1.583 | 7.540 | 3.83E-05 | 1.04E-03 | -0.82 | 0.44 | 0.76 | 0.44 | 1.58 |
| hsa-miR-204-3p | 1.640 | 10.765 | 9.17E-04 | 9.09E-03 | -1.49 | 0.44 | 0.15 | 0.90 | 1.64 |
| **hsa-miR-223-3p** | **-2.777** | **10.433** | **3.38E-06** | **3.06E-04** | **2.91** | **0.67** | **0.13** | **0.53** | **2.78** |
| hsa-miR-22-3p | 2.269 | 10.946 | 7.11E-03 | 3.96E-02 | -1.73 | 0.63 | 0.54 | 1.81 | 2.27 |
| hsa-miR-25-3p | 1.557 | 8.101 | 2.18E-03 | 1.73E-02 | -0.90 | 0.24 | 0.65 | 1.06 | 1.56 |
| hsa-miR-26b-5p | -2.001 | 7.551 | 5.51E-04 | 6.56E-03 | 3.26 | 0.62 | 1.26 | 0.95 | 2.00 |
| **hsa-miR-30c-1-3p** | **2.151** | **8.687** | **3.92E-06** | **3.06E-04** | **-1.65** | **0.38** | **0.51** | **0.57** | **2.15** |
| hsa-miR-3149 | -1.871 | 8.478 | 2.30E-06 | 2.53E-04 | 0.59 | 0.19 | -1.29 | 0.54 | 1.87 |
| hsa-miR-3158-5p | 2.513 | 11.687 | 3.31E-05 | 1.00E-03 | -2.00 | 0.49 | 0.51 | 0.91 | 2.51 |
| hsa-miR-3162-3p | 1.984 | 7.095 | 6.93E-05 | 1.64E-03 | -1.21 | 0.22 | 0.78 | 0.87 | 1.98 |
| hsa-miR-3178 | 2.004 | 9.584 | 7.33E-04 | 7.96E-03 | -1.97 | 0.65 | 0.04 | 0.98 | 2.00 |
| hsa-miR-3182 | -1.855 | 11.441 | 2.02E-04 | 3.42E-03 | 1.73 | 0.42 | -0.12 | 0.83 | 1.86 |
| hsa-miR-3195 | 1.795 | 7.364 | 5.38E-06 | 3.68E-04 | -1.50 | 0.18 | 0.30 | 0.57 | 1.80 |
| hsa-miR-3202 | 2.255 | 10.403 | 1.68E-03 | 1.42E-02 | -1.47 | 0.24 | 0.78 | 1.52 | 2.25 |
| **hsa-miR-320a** | **1.895** | **8.023** | **2.84E-03** | **2.07E-02** | **-1.39** | **0.33** | **0.51** | **1.35** | **1.90** |
| hsa-miR-320b | 2.085 | 8.241 | 1.93E-03 | 1.57E-02 | -1.61 | 0.25 | 0.48 | 1.42 | 2.08 |
| hsa-miR-320c | 1.985 | 7.687 | 2.61E-03 | 1.98E-02 | -1.44 | 0.34 | 0.55 | 1.39 | 1.98 |
| hsa-miR-320d | 1.757 | 6.567 | 4.21E-03 | 2.69E-02 | -1.11 | 0.34 | 0.65 | 1.32 | 1.76 |
| hsa-miR-320e | 1.687 | 5.872 | 3.44E-03 | 2.34E-02 | -1.21 | 0.17 | 0.48 | 1.26 | 1.69 |
| **hsa-miR-32-3p** | **-2.213** | **9.674** | **2.63E-05** | **9.00E-04** | **0.65** | **0.21** | **-1.56** | **0.87** | **2.21** |
| hsa-miR-339-5p | 1.687 | 6.962 | 2.42E-03 | 1.86E-02 | -1.30 | 0.29 | 0.39 | 1.16 | 1.69 |
| hsa-miR-3591-5p | -1.511 | 6.839 | 1.36E-08 | 1.42E-05 | 0.65 | 0.11 | -0.86 | 0.20 | 1.51 |
| hsa-miR-3607-3p | -1.853 | 6.927 | 7.39E-06 | 4.67E-04 | 0.63 | 0.31 | -1.22 | 0.55 | 1.85 |
| hsa-miR-3607-5p | -1.508 | 6.302 | 3.55E-06 | 3.06E-04 | 1.25 | 0.33 | -0.26 | 0.30 | 1.51 |
| hsa-miR-3609 | -1.542 | 5.591 | 9.60E-07 | 1.43E-04 | 1.36 | 0.34 | -0.18 | 0.19 | 1.54 |
| hsa-miR-361-3p | 1.565 | 7.592 | 1.29E-04 | 2.49E-03 | -0.56 | 0.23 | 1.00 | 0.71 | 1.56 |
| hsa-miR-3621 | 1.806 | 11.431 | 2.26E-05 | 8.42E-04 | -1.96 | 0.43 | -0.16 | 0.54 | 1.81 |
| hsa-miR-3654 | -1.939 | 7.955 | 4.80E-08 | 2.37E-05 | 0.39 | 0.12 | -1.55 | 0.37 | 1.94 |
| hsa-miR-423-3p | 2.081 | 7.928 | 4.69E-03 | 2.90E-02 | -1.64 | 0.21 | 0.44 | 1.64 | 2.08 |
| hsa-miR-4258 | 2.034 | 8.581 | 2.12E-05 | 8.19E-04 | -0.56 | 0.29 | 1.47 | 0.74 | 2.03 |
| hsa-miR-4284 | -2.957 | 10.949 | 3.56E-05 | 1.04E-03 | 2.98 | 0.81 | 0.02 | 0.88 | 2.96 |
| hsa-miR-4306 | 2.273 | 11.457 | 4.36E-03 | 2.74E-02 | -1.42 | 0.20 | 0.85 | 1.78 | 2.27 |
| hsa-miR-4317 | 2.177 | 7.498 | 1.10E-05 | 5.45E-04 | -0.81 | 0.18 | 1.36 | 0.78 | 2.18 |
| hsa-miR-4449 | 2.358 | 7.820 | 5.05E-05 | 1.32E-03 | -2.12 | 0.33 | 0.24 | 0.97 | 2.36 |
| hsa-miR-4455 | -1.674 | 10.015 | 6.17E-05 | 1.51E-03 | 0.47 | 0.17 | -1.21 | 0.72 | 1.67 |
| hsa-miR-4458 | -1.541 | 5.749 | 1.52E-05 | 6.59E-04 | 0.36 | 0.18 | -1.18 | 0.54 | 1.54 |
| hsa-miR-4463 | 1.610 | 6.321 | 2.63E-04 | 4.13E-03 | -1.26 | 0.16 | 0.35 | 0.83 | 1.61 |
| hsa-miR-4484 | 2.002 | 10.469 | 5.08E-04 | 6.17E-03 | -2.00 | 0.52 | 0.00 | 1.01 | 2.00 |
| hsa-miR-4497 | 1.741 | 10.535 | 1.93E-03 | 1.57E-02 | -1.77 | 0.48 | -0.03 | 1.08 | 1.74 |
| hsa-miR-4516 | 1.632 | 13.518 | 4.98E-04 | 6.14E-03 | -0.97 | 0.36 | 0.66 | 0.85 | 1.63 |
| hsa-miR-4532 | 1.836 | 12.312 | 2.29E-03 | 1.78E-02 | -1.81 | 0.48 | 0.03 | 1.19 | 1.84 |
| hsa-miR-4534 | 2.278 | 9.550 | 3.31E-05 | 1.00E-03 | -1.80 | 0.38 | 0.48 | 0.86 | 2.28 |
| hsa-miR-4540 | -1.547 | 7.754 | 1.06E-03 | 1.01E-02 | 0.58 | 0.14 | -0.97 | 0.97 | 1.55 |
| hsa-miR-4640-3p | 1.668 | 6.510 | 4.11E-06 | 3.06E-04 | -1.05 | 0.21 | 0.62 | 0.49 | 1.67 |
| hsa-miR-4687-3p | 2.421 | 9.278 | 6.64E-04 | 7.48E-03 | -2.05 | 0.53 | 0.38 | 1.33 | 2.42 |
| hsa-miR-4732-3p | -1.772 | 8.778 | 4.21E-04 | 5.61E-03 | 0.36 | 0.59 | -1.41 | 0.75 | 1.77 |
| hsa-miR-4741 | 1.666 | 7.123 | 2.85E-04 | 4.41E-03 | -1.44 | 0.36 | 0.23 | 0.79 | 1.67 |
| hsa-miR-4792 | 1.624 | 7.548 | 2.93E-03 | 2.11E-02 | -1.59 | 0.31 | 0.04 | 1.15 | 1.62 |
| **hsa-miR-483-5p** | **1.846** | **9.680** | **8.66E-04** | **8.76E-03** | **-1.37** | **0.39** | **0.47** | **1.06** | **1.85** |
| hsa-miR-491-3p | -2.889 | 11.997 | 4.41E-04 | 5.68E-03 | 0.79 | 1.43 | -2.09 | 0.45 | 2.89 |
| hsa-miR-519e-5p | 1.674 | 8.344 | 5.25E-04 | 6.33E-03 | -1.53 | 0.23 | 0.14 | 0.93 | 1.67 |
| **hsa-miR-542-5p** | **2.211** | **7.834** | **2.19E-05** | **8.30E-04** | **-1.84** | **0.42** | **0.37** | **0.75** | **2.21** |
| hsa-miR-5681b | -1.513 | 10.302 | 1.62E-03 | 1.39E-02 | 0.33 | 0.54 | -1.19 | 0.82 | 1.51 |
| hsa-miR-5684 | -1.710 | 10.170 | 2.84E-03 | 2.07E-02 | 0.79 | 0.18 | -0.92 | 1.24 | 1.71 |
| hsa-miR-5701 | -3.127 | 7.568 | 3.85E-06 | 3.06E-04 | 0.52 | 0.76 | -2.61 | 0.63 | 3.13 |
| hsa-miR-574-5p | -1.552 | 8.377 | 7.64E-07 | 1.31E-04 | 0.52 | 0.16 | -1.03 | 0.37 | 1.55 |
| hsa-miR-631 | 1.625 | 6.412 | 2.53E-05 | 8.98E-04 | -1.74 | 0.19 | -0.11 | 0.61 | 1.63 |
| hsa-miR-642a-3p | 2.530 | 7.403 | 1.16E-04 | 2.30E-03 | -2.13 | 0.40 | 0.40 | 1.15 | 2.53 |
| hsa-miR-642b-3p | 2.087 | 6.915 | 2.10E-03 | 1.67E-02 | -1.97 | 0.15 | 0.11 | 1.46 | 2.09 |
| hsa-miR-664b-5p | -1.702 | 7.667 | 9.21E-04 | 9.09E-03 | 0.75 | 0.25 | -0.95 | 1.03 | 1.70 |
| **hsa-miR-675-5p** | **2.323** | **10.788** | **3.75E-05** | **1.04E-03** | **-1.85** | **0.46** | **0.48** | **0.84** | **2.32** |
| hsa-miR-711 | 1.826 | 8.524 | 1.03E-04 | 2.10E-03 | -1.72 | 0.38 | 0.10 | 0.76 | 1.83 |
| hsa-miR-99a-5p | 1.964 | 7.534 | 8.55E-04 | 8.70E-03 | -1.24 | 0.50 | 0.73 | 1.09 | 1.96 |
| hsa-miR-99b-5p | 1.706 | 6.788 | 8.79E-04 | 8.81E-03 | -1.06 | 0.28 | 0.65 | 1.01 | 1.71 |
| hsa-miRPlus-A1015 | -2.028 | 7.871 | 5.68E-08 | 2.37E-05 | 0.23 | 0.23 | -1.80 | 0.34 | 2.03 |
| hsv1-miR-H18 | 1.667 | 7.439 | 2.58E-05 | 8.98E-04 | -1.94 | 0.29 | -0.27 | 0.58 | 1.67 |
| hsv1-miR-H6-5p | 1.524 | 6.885 | 8.69E-03 | 4.55E-02 | -1.74 | 0.20 | -0.21 | 1.32 | 1.52 |
| SNORD10 | -2.698 | 6.775 | 6.47E-07 | 1.31E-04 | 2.30 | 0.61 | -0.39 | 0.32 | 2.70 |
| SNORD38B | -1.553 | 7.012 | 6.07E-04 | 6.99E-03 | 1.76 | 0.46 | 0.21 | 0.75 | 1.55 |
| SNORD44 | -2.191 | 7.219 | 7.95E-09 | 1.42E-05 | 1.46 | 0.18 | -0.73 | 0.31 | 2.19 |
| SNORD49A | -1.501 | 5.655 | 9.53E-05 | 2.05E-03 | 1.37 | 0.57 | -0.14 | 0.27 | 1.50 |
| SNORD4A | -2.226 | 7.048 | 4.92E-08 | 2.37E-05 | 0.87 | 0.30 | -1.35 | 0.31 | 2.23 |
| SNORD68 | -1.932 | 9.858 | 8.52E-03 | 4.52E-02 | 1.16 | 0.35 | -0.77 | 1.65 | 1.93 |

In bold, miRNAs analyzed by qPCR
