# Supplementary figures and images for: MiRNA profiling of whole trabecular bone: identification of osteoporosis-related changes in MiRNAs in human hip bones
Source: BMC Med Genomics. 2015 Nov 10;8:75. doi: 10.1186/s12920-015-0149-2 (PMC4640351; doi:10.1186/s12920-015-0149-2)

CD3 Gene Expression


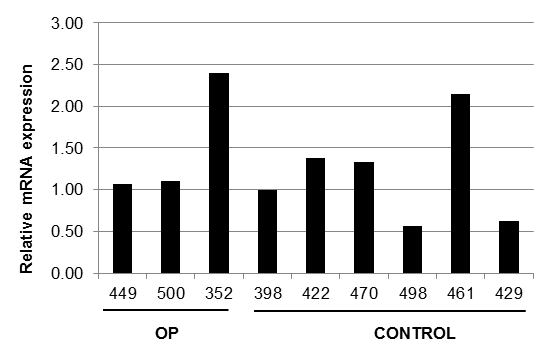


**Relative mRNA expression**

B

A

A

Figure S1

Supplement: Additional file 2: Figure S1. — Gene expression quantification of CD3 marker by Real-Time PCR in total bone samples. OP samples (n = 3); Osteoarthritic (control) samples (n = 6). Results are expressed as mean of relative expression and standard deviation. A) Gene expression in each bone sample; B) Gene expression comparison between biological groups. (DOCX 59 kb) [file 12920_2015_149_MOESM2_ESM.docx]
